# Land‐use affects pollinator‐specific resource availability and pollinator foraging behaviour

**DOI:** 10.1002/ece3.11061

**Published:** 2024-03-07

**Authors:** Markus Birkenbach, Florian Straub, Anna Kiesel, Manfred Ayasse, Lena Wilfert, Jonas Kuppler

**Affiliations:** ^1^ Institute of Evolutionary Ecology and Conservation Genomics Ulm University Ulm Germany

**Keywords:** anthropogenic influence, bumblebee, pollination services, pollinator‐plant interaction, syrphid fly

## Abstract

Land‐use management is a key factor causing pollinator declines in agricultural grasslands. This decline can not only be directly driven by land‐use (e.g., habitat loss) but also be indirectly mediated through a reduction in floral resource abundance and diversity, which might in turn affect pollinator health and foraging. We conducted surveys of the abundance of flowering plant species and behavioural observations of two common generalist pollinator species, namely the bumblebee *Bombus lapidarius* and the syrphid fly *Episyrphus balteatus*, in managed grasslands of variable land‐use intensity (LUI) to investigate whether land‐use affects (1) resource availability of the pollinators, (2) their host plant selection and (3) pollinator foraging behaviour. We have found that the floral composition of plant species that were used as resource by the investigated pollinator species depends on land‐use intensity and practices such as mowing or grazing. We have also found that bumblebees, but not syrphid flies, visit different plants depending on LUI or management type. Furthermore, LUI indirectly changed pollinator behaviour via a reduction in plot‐level flower diversity and abundance. For example, bumblebees show longer flight durations with decreasing flower cover indicating higher energy expenditure when foraging on land‐use intensive plots. Syrphid flies were generally less affected by local land use, showing how different pollinator groups can differently react to land‐use change. Overall, we show that land‐use can change resource composition, abundance and diversity for pollinators, which can in turn affect pollinator foraging behaviour and potentially contribute to pollinator decline in agricultural grasslands.

## Graphical abstract

We investigated whether floral resource availability and foraging behaviour of two common pollinator species, *Bombus lapidarius* and *Episyrphus balteatus*, change with land‐use intensity and land‐use practices in agricultural grasslands. We were able to show that, for both pollinators, the composition of potential host plants changed with land‐use intensity and management practices. Furthermore, land‐use indirectly affected pollinator behaviour by reducing plot‐level flower cover and diversity, potentially decreasing the pollinators' foraging efficiency.

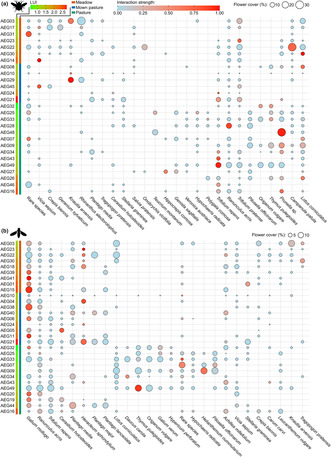

## 1 INTRODUCTION

Insects are essential for the pollination of the majority of wild flowering plants and crops and therefore for the preservation of functioning ecosystems and for ensuring food production (Klein et al., 2007; Ollerton et al., 2011). Pollination is however threatened by declining pollinator diversity and abundances (Seibold et al., 2019; Wagner et al., 2021). The reasons for these declines are manifold, for example land‐use intensification and its associated limitation of floral resources, habitat degradation and the use of pesticides (Goulson et al., 2015; Potts et al., 2010). In agricultural grasslands, land‐use intensification can alter the abundance, diversity and composition of plants because of fertilisation and intensive grazing or mowing, leading to species‐poor communities with a low abundance of floral resources for pollinators (Binkenstein et al., 2013; Socher et al., 2013). This may cause nutritional stress for pollinators and eventually reduce pollinator health or change foraging behaviour (Woodard & Jha, 2017). Additionally, shifts in plant–pollinator interaction patterns might lead to plant–pollinator mismatches (Peters et al., 2022; Weiner et al., 2011) and ultimately to parallel declines of plants and pollinators (Biesmeijer et al., 2006). In such a scenario, pollinators might aim to exploit the remaining floral resources by optimising flower visitation patterns.

Optimal foraging theory predicts that pollinators should maximise foraging efficiency, that is their net energy gain during foraging (Heinrich, 1975; Pyke, 1984). Foraging efficiency of pollinators can be determined by elements of their foraging behaviour such as flower handling times, flight durations or flower constancy (Pyke & Starr, 2021; Willmer, 2011). Flower constancy describes the likelihood that a pollinator keeps visiting flowers of the same plant species rather than switching to another species within a foraging trip. It is a strategy that reduces foraging complexity, for example, by not having to learn the handling of new flowers (Chittka et al., 1999; Hayes & Grüter, 2023). At the same time, high flower constancy is also beneficial for the visited plants, as it increases pollination success by decreasing the deposition of heterospecific pollen (Waser, 1986).

Foraging behaviour varies between individual pollinators and is dependent on the plant species visited (Willmer, 2011), but it can also be affected by land‐use‐induced limitation of floral resources. For example, food shortage can decrease foraging efficiency in honey bees by reducing flight performance (Brodschneider et al., 2022) or foraging activity (Scofield & Mattila, 2015). Additionally, poor nutrition can affect pollinator health, for example by reducing pollinator body size, which is directly linked to foraging behaviour and vice versa (Chole et al., 2019; Klumpers et al., 2019; Straub et al., 2023). Land‐use and its practices shape the abundance and composition of plants and thus the availability and quality of floral rewards (Busch et al., 2019). This can influence the pollinator's choice of which plant to visit, and therefore drive plant–pollinator interaction patterns in agricultural landscapes (Fowler et al., 2016; Ruedenauer et al., 2016; Vaudo et al., 2015). Moreover, climatic variables such as temperature are important predictors of pollinator behaviour (McCallum et al., 2013), with different pollinator groups having different thermal optima (Kühsel & Blüthgen, 2015). Bumblebees, for example, are active at relatively low air temperatures compared to other pollinators due to their body size and thermoregulation (Kenna et al., 2021). Therefore, land‐use and the associated reduction of floral resources might, together with climatic factors, determine the pollinators' efficiency in foraging and ultimately pollination in agricultural areas.

Bumblebees and syrphid flies are two important pollinator groups whose abundance has been relatively robust to land‐use intensification (Weiner et al., 2014). They represent two extreme pollinator types: bumblebees are social insects with a local nesting site, whereas syrphids are solitary flies. Thus, both pollinators might react differently to land‐use‐induced changes in floral resource availability. Bumblebees are large‐bodied central place foragers that visit a variety of different plant species, for example from the Asteraceae, Fabaceae or Lamiaceae family (Fründ et al., 2010; Sikora & Kelm, 2012), in order to collect pollen and nectar for themselves or to provide food for their larvae (Goulson, 2010). Foraging distances of bumblebees range from several hundred metres up to estimates of 2–3 km from the nesting site depending on individual body size and colony size, as well as resource availability in the landscape (Knight et al., 2005; Westphal et al., 2006). In contrast to bees, syrphids rely on floral resources exclusively during their adult stage, since their larvae feed on other insects, faeces or plant material (Doyle et al., 2020). They visit a high variety of host plants with preferences for open, yellow or white flowers, including species from, for example the Ranunculaceae or Apiaceae family (Cowgill et al., 1993; Dunn et al., 2020), potentially complementing the provision of the pollination services of bees. Furthermore, hoverflies are migratory insects that can transport pollen over long distances of up to several hundred kilometres, but usually forage within one food patch when breeding (Doyle et al., 2020; Wotton et al., 2019). During the reproduction stage, syrphid flies can show varying foraging distances and dispersal rates depending on competition, floral resource and larval host abundance (Almohamad et al., 2009), but they are commonly considered to be higher compared to bees (Doyle et al., 2020; MacLeod, 1999; Rader et al., 2011).

In this study, we investigated the effect of LUI on the resource availability and behaviour of two common generalist pollinators: the social bumblebee *Bombus lapidarius* (Linnaeus, 1758) and the syrphid fly *Episyrphus balteatus* (De Geer, 1776). As study site, we chose one of the German Biodiversity Exploratories, namely the Schwäbische Alb in south‐western Germany. The grassland plots in the Biodiversity Exploratories are managed by the local farmers and can vary from extensively used pastures to intensive grasslands that are mown and fertilised several times per year, creating a characteristic land‐use gradient over all sites (Fischer et al., 2010). We conducted surveys of local flowering plant species abundance and tracked the behaviour of individual pollinators by recording the plant species visited, the time spent on a plant, the number of flowers visited per plant and the duration and distances of flights between plants in order to answer the following questions:

1. Are composition, diversity or abundance of plants used as floral resource by the respective pollinator species dependent on LUI and management practices; and if so, are these changes reflected in the observed plant–pollinator interaction patterns?
2. Does LUI, either directly or indirectly through a change in floral resources, affect pollinator behaviour in the field?
3. Are the two pollinator groups affected differently by LUI and associated floral resource change?

## 2 MATERIALS AND METHODS

### 2.1 Study design

**FIGURE A1:**
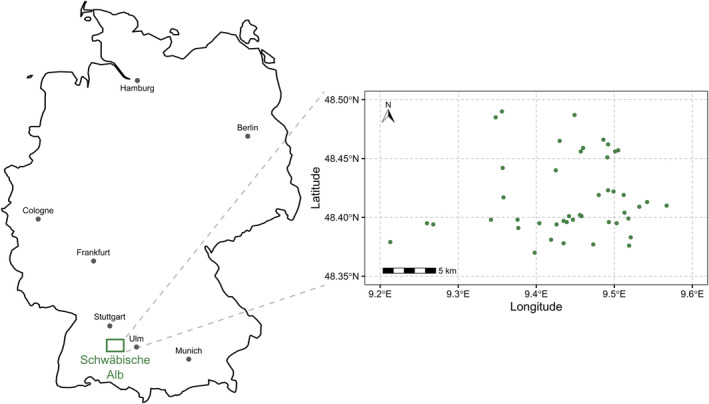
Location of the study area Schwäbische Alb within Germany (left) and its extension with approximate position of the 43 study plots (right).

The study was conducted from the 14th of June until the 29th of July 2021 on grassland plots (50 × 50 m) within the Biodiversity Exploratories in the Schwäbische Alb region (Figure A1; 48°43′ N, 9°37′ E, 460–860 m above mean sea level) in south‐western Germany (Fischer et al., 2010). We surveyed all 50 experimental grassland plots (EPs) for individuals of the red‐tailed bumblebee (*B. lapidarius*) and the marmalade syrphid fly (*E. balteatus*) and conducted observations of their foraging behaviour. Both pollinator species are highly abundant in Germany and are not considered endangered (Ssymank et al., 2011; Westrich et al., 2011). Previous studies have shown that they are also common within the sites of the Biodiversity Exploratories (Weiner et al., 2014). Some plots were visited repeatedly (see Table A1) to maximise sample size of behavioural observations, while keeping the period between repeated visitations as short as possible (the maximum difference between first and last visit of a plot was 35 days). Additionally, we assessed the total plot flower cover and individual cover of flowering herbaceous plant species in order to calculate plot‐level flower diversity (see details below). Land‐use and climate data were obtained from the Biodiversity Exploratories Information system BExIS (http://doi.org/10.17616/R32P9Q).

### 2.2 Plot‐related variables

The EPs of the Biodiversity Exploratories are embedded in real‐world management, meaning that they are handled by local owners and farmers according to their own needs. The land‐use intensity index (LUI) is calculated yearly by combining the three components fertilisation (kg N ha^−1^ year^−1^), mowing (cuts year^−1^) and grazing (livestock unit‐grazing days·ha^−1^ year^−1^) (Blüthgen et al., 2012) based on questionnaires with the local land owners (Vogt et al., 2019). Thus, LUI represents a natural land‐use gradient, which can range from highly extensive plots that are, for example grazed by sheep for only a few days annually to intensive meadows that are heavily fertilised and mown several times per year. In 2021, the LUI on EPs in the Schwäbische Alb region ranged from 0.58 to 2.75, where a higher LUI represents a higher land‐use intensity (Table A1). To calculate LUI, each of the LUI components was standardised relative to its mean within the Schwäbische Alb region using the LUI calculation tool (Ostrowski et al., 2020) implemented in BExIS. Furthermore, the plots were characterised as three land‐use management types, namely meadows, pastures and mown pastures. Meadows refer to plots from which biomass was removed exclusively by mowing, whereas pastures were exclusively grazed; mown pastures showed a mixture of both practices.

Total flower cover (percentage of area covered by flowers compared to e.g., grasses) on the whole 50 × 50 m plot was estimated visually in four categories (<1%, 1%–10%, 11%–40%, >40%) following Neumüller et al. (2020). Additionally, we assessed the identity of all flowering herbaceous plant species and estimated their individual contribution to the total flower cover (percentage of individual plant species flower cover compared with all flowers present) in the same four categories. Unknown plant species were identified with the help of the Flora Incognita app (Mäder et al., 2021). Assessment of flower cover and composition was repeated after revisiting the same plot later in the season, since they might have changed over time. Air temperature (at 2 m above ground) was measured by standardised climate stations located on each EP of the Exploratories and obtained from BExIS (dataset ID 19007).

### 2.3 Behavioural observations

Behavioural observations in the field were always conducted by the same two persons via a Pocketobserver using the software Observer XT v15 by Noldus (Leesburg, VA, USA). One person followed one pollinator at a time and recorded the following behavioural variables: (1) the plant species visited by the pollinator, (2) the time spent on a plant individual, (3) the number of floral units visited per plant individual (4) the flight duration, that is the time that pollinators took to fly from one plant to another and (5) the flight distance. We counted Asteraceae flower heads and secondary umbels of Apiaceae as single floral units according to Dicks et al. (2002), as pollinators can walk in between individual flowers and do not have to fly. Durations (variables 2 and 4) were automatically tracked by the observational software after manually selecting the behaviour of the observed pollinator, that is plant visitation or foraging flight. Number of floral units visited (3) and flight distances (5) were estimated in categories, since it was not possible to accurately count or measure these variables during an observation (Table A2). The handling time of an individual floral unit was calculated by dividing the time spent on a plant individual by the mean number of floral units visited (see Methodology of mean calculation of categorical variables in Appendix 1).

We stayed on a plot for a maximum of 2 h and observed as many individuals of the target pollinator species as possible before switching sites. The visitation of a plot was only abandoned if none of the target species was found within 30 min, or the weather conditions were not met (raining and/or below 15°C). Individual observations were stopped when the observed individual pollinator moved out of sight or when it left the plot. The mean duration of an observation was 184 ± 110 s for bumblebees and 314 ± 184 s for syrphid flies.

### 2.4 Statistical analysis

#### 2.4.1 Plant composition and plant–pollinator interactions

All analyses were performed separately for bumblebees and syrphid flies by using the software R version 4.2.2 (R Core Team, 2022). We used PERMANOVA to analyse whether differences in both plant composition and the observed plant–pollinator interactions are dependent on LUI and the three management types (meadow, mown pasture and pasture). When analysing plant composition, we only considered plant species that were visited by any individual of the respective pollinator species throughout the study (Tables A3 and A4) to exclude plants that are not used as potential floral resource. Species with very low abundance, that is when the combined flower cover over all plots was <10%, were grouped together as a category of ‘rare species’. For the analysis of plant–pollinator interactions, the number of interactions with each plant species was standardised with the total number of interactions per plot; the standardised value was referred to as the ‘interaction strength’ and ranged from 0 to 1 (low to high interaction strength; see Tables A3 and A4 for average interaction strengths with individual plant species). We excluded plots for which the total number of plant–pollinator interactions was lower than 10 (excluded plots: bumblebees: none; syrphid flies: *N* = 4). We calculated Bray–Curtis similarity distance matrices based on both the individual flower cover of each plant species (see Methodology of mean calculation of categorical variables in Appendix 1) and interaction strength and performed permutational multivariate analysis of variance (PERMANOVA, 9999 permutations) by using the *adonis2* function of the *vegan* package (Oksanen et al., 2022).

We created bubble heatmaps for a two‐dimensional visualisation of flower cover, represented by bubble size, and interaction strength, represented by a colour gradient using *ggplot2* (Wickham, 2016). The order of plant species along the horizontal axis was extracted from a cluster analysis based on similarities in individual plant species flower cover by using the package *pheatmap* (Kolde, 2019). The order of plots along the vertical axis was manually arranged by management types and LUI.

#### 2.4.2 Calculation of variables for analyses of foraging behaviour

Flower diversity and flower cover on the plot were calculated separately for bumblebees and syrphid flies, again only considering plant species that were visited by the respective pollinators. We used the Shannon Index as a measurement of diversity, which was calculated with the *vegan* package based on the flower cover of individual plant species (see Methodology of mean calculation of categorical variables in Appendix 1) (Oksanen et al., 2022). Air temperature was recorded at local climate stations, for which we calculated means across 1 h to compensate for variations (e.g., passing clouds). Floral constancy was calculated by dividing the number of switches between the different plant species by the total number of plant individuals visited during an observation of an individual pollinator and resulted in values ranging from 0 to 1. For the analysis of flower constancy, we excluded observations if the total number of interactions with plant individuals was lower than three (bumblebees: 27 excluded individuals; syrphid flies: 58 excluded individuals).

#### 2.4.3 Foraging behaviour

We conducted linear mixed models (LMM) with the package *lme4* (Bates et al., 2015) to explore whether LUI, management types, flower cover, plant diversity or temperature had a general effect on behavioural response variables. Plant species was included as a predictor when analysing behaviours regarding the handling of flowers, namely time spent on a plant individual, number of flowers visited per plant and flower handling time. ‘Pollinator individual’ nested within ‘plot’ was used as a random factor.

We performed structural equation modelling (SEM) to discover possible indirect effects of LUI via flower diversity and flower cover and direct effects of temperature on behavioural variables by using the *piecewiseSEM* package (Lefcheck, 2016). We employed flower constancy, time spent on plant individuals and flight duration as behavioural response variables, as they represent the most accurate measurements and result in the best model fits compared with categorical variables. We used means of the behavioural variables across pollinator individuals to eliminate ‘pollinator individual’ as a random factor, with only ‘plant species’ and ‘plot’ as random factors to simplify the models. If a pollinator individual visited multiple plant species during an observation, plant species was assigned to a new category, namely ‘mixed species’. Since floral constancy was high for both species (67% of bumblebees and 65% of syrphid flies showed complete foraging constancy), we fitted generalised linear mixed models (GLMM) with correction for zero‐inflation by using the *glmmTMB* package (Brooks et al., 2017). For the visualisation of the SEMs, the estimates of flower constancy were inverted to show the true effects (1 = totally constant, 0 = totally inconstant). All variables were tested for collinearity by using Pearson linear correlations prior to running individual models (exclusion criteria |*r*| > .7) and no predictors needed to be excluded. All models were validated using the *check_model* function from the *performance* package (Lüdecke et al., 2021).

## 3 RESULTS

**FIGURE A2:**
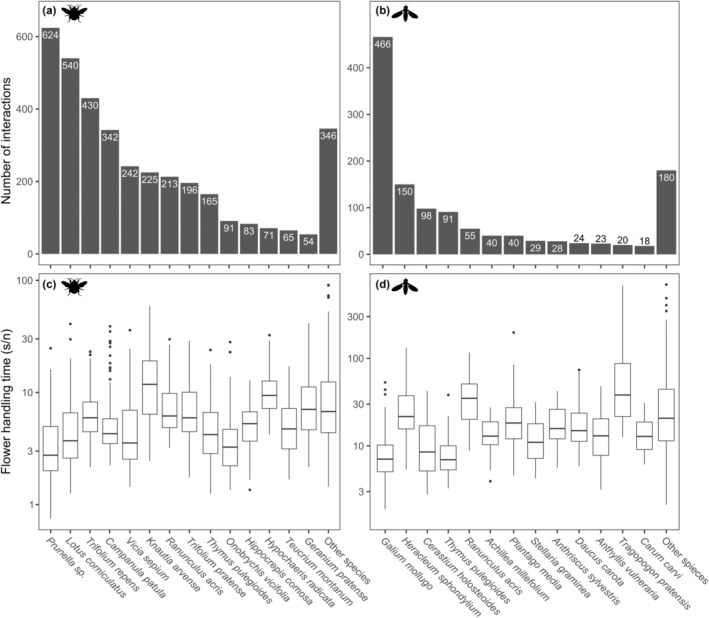
Number of interactions of the bumblebee *Bombus lapidarius* (a) and the syrphid fly *Episyrphus balteatus* (b) with their most visited plant species and their respective flower handling times (c, d).

We observed 307 bumblebee workers (*B. lapidarius*) and 315 syrphid fly individuals (*E. balteatus*) on a total of 43 out of the 50 plots in the Schwäbische Alb region. Bumblebees were observed on 27 plots and syrphid flies on 34 plots with an overlap of 16 plots (Table A1). A total of 3699 plant–pollinator interactions were recorded for bumblebees and 1271 interactions for syrphid flies (Figure A2, Tables A3 and A4).

### 3.1 Species composition of plants visited by the pollinators

**FIGURE 1:**
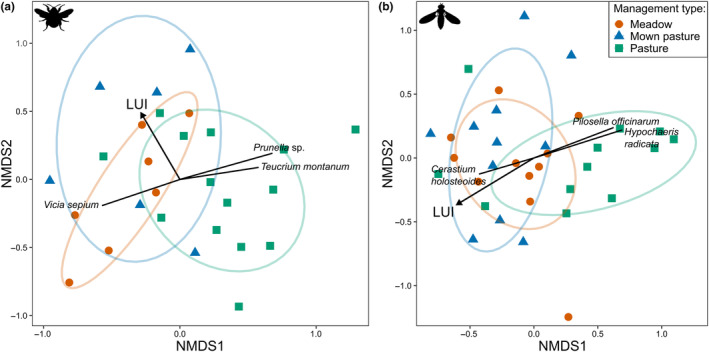
Composition of plant species visited by the bumblebee *Bombus lapidarius* (a: *N* = 27) and the syrphid fly *Episyrphus balteatus* (b: *N* = 34) on two dimensions by non‐metric multidimensional scaling (NMDS) based on Bray–Curtis similarities of flower cover of individual plant species. Every point represents the composition of plant species on one plot. The plots were separated by the management types meadow (red), mown pasture (blue) and pasture (green) and LUI (black arrow). LUI and plant species contributing most to the separation of the plots are depicted as vectors. Stress‐level A: 0.208; Stress‐level B: 0.225.

Plant composition of species visited by the two pollinators significantly differed among grassland management types (Bumblebees: PERMANOVA_Management_: *R* ^2^ = .16, Pseudo‐*F*_2,27_ = 2.42, *p* = <.001, Figure 1a; Syrphid flies: PERMANOVA_Management_: *R* ^2^ = .10, Pseudo‐*F*_2,34_ = 1.86, *p* = .011, Figure 1b) and along a LUI gradient (Bumblebees: PERMANOVA_LUI_: *R* ^2^ = .07, Pseudo‐*F*_1,27_ = 1.97, *p* = .019, Figure 1a; Syrphid flies: PERMANOVA_LUI_: *R* ^2^ = .06, Pseudo‐*F*_2,34_ = 2.11, *p* = .016, Figure 1b). *R* ^2^ and *p*‐values indicated a higher dependence on management types than LUI. For both pollinator species, pairwise PERMANOVA comparisons showed that un‐mown pastures differed significantly from meadows (bumblebees: *p* = .003; syrphid flies: *p* = .022) and mown pastures (bumblebees: *p* = .013; syrphid flies: *p* = .022), but meadows did not differ from mown pastures (bumblebees: *p* = .348; syrphid flies: *p* = .930). Plant species associated with pastures and significantly contributing to this separation included, for example *Prunella* sp. or *Teucrium montanum* for bumblebees and *Hypochaeris radicata* or *Pilosella officinarum* for syrphid flies. In contrast, *Vicia sepium* (bumblebees) and *Cerastium holosteoides* (syrphid flies) were plant species associated with mown plots (Figure 1, Table A5).

### 3.2 Plant–pollinator interactions

**FIGURE 2:**
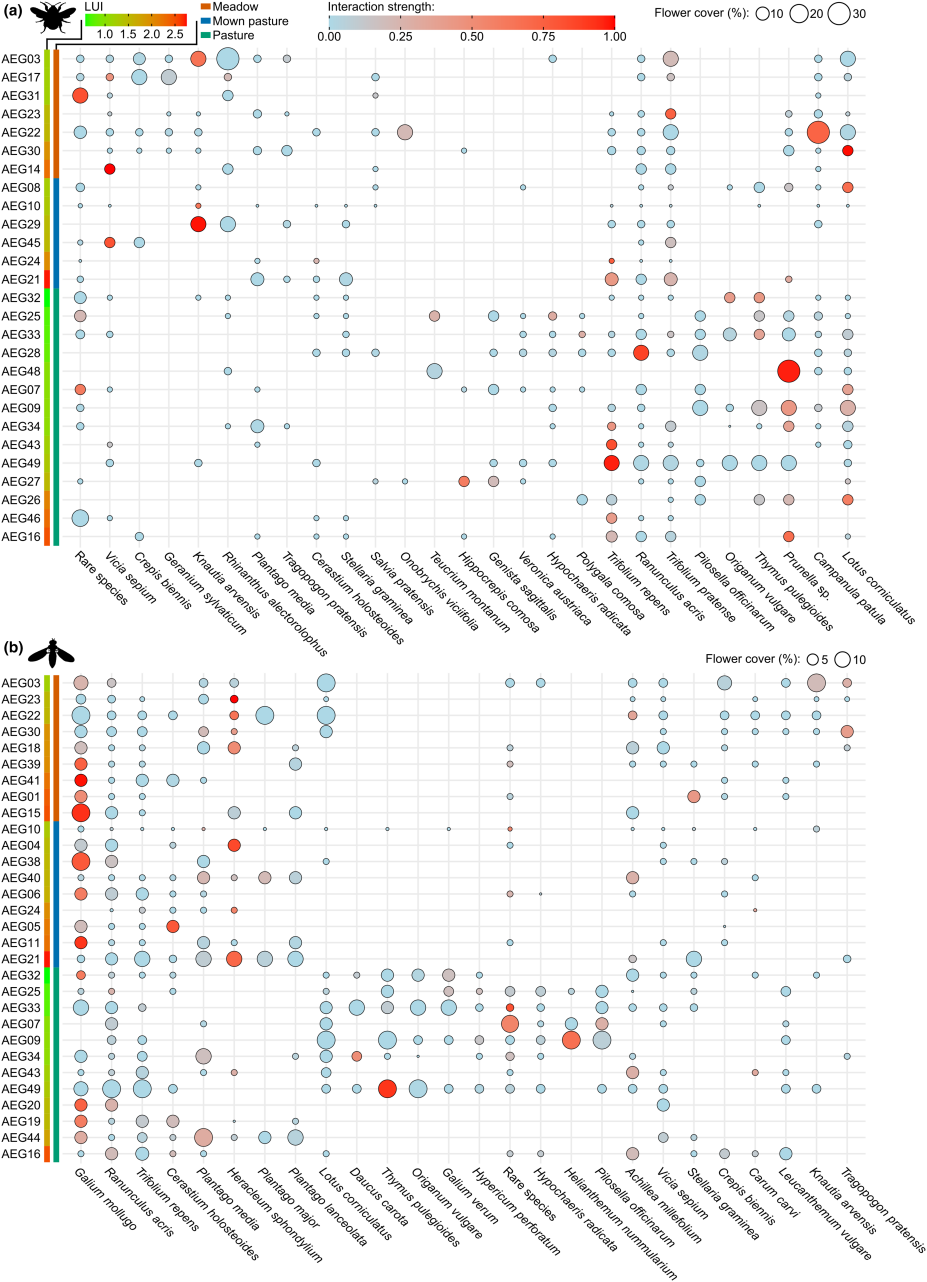
Bubble heatmap visualising the flower cover of individual plant species (area) and strength of interaction of the bumblebee *Bombus lapidarius* (a) and the syrphid fly *Episyrphus balteatus* (b) with the respective plant species (colour). Interaction strength is the number of interactions with plant species standardised by the total number of interactions per plot: Light blue shows low interaction strength and red high interaction strength. The vertical axis was arranged by the management types, namely meadow (red), mown pasture (blue) and pasture (green), and afterwards along a LUI gradient; the horizontal axis was clustered by similarities in flower cover of plant species. Plant species clustered together in association with management types, for example for bumblebees, plant species ranging from *Vicia sepium* to *Rhinanthus alectorolophus* were mainly present in meadows, with species ranging from *Pilosella officinarum* to *Prunella* sp. occurring in pastures, whereas others, such as *Ranunculus acris*, *Trifolium pratense* and *Trifolium repens*, were equally present on many plots (compare Table A5). Plant species with low abundance are grouped together as ‘rare species’.

After finding differences in compositions of visited plant species, we investigated whether the observed interactions of the pollinators with plant species differed with management type or LUI. We found that the interaction pattern of bumblebees significantly differed with management type (PERMANOVA_Management_: *R* ^2^ = .11, Pseudo‐*F*_2,27_ = 1.58, *p* = .047) and LUI (PERMANOVA_LUI_: *R* ^2^ = .07, Pseudo‐*F*_1,27_ = 1.90, *p* = .03, Figure 2a), but not for syrphid flies (PERMANOVA_Management_: *R* ^2^ = .07, Pseudo‐*F*_2,34_ = 1.18, *p* = .27; PERMANOVA_LUI_: *R* ^2^ = .02, Pseudo‐*F*_1,34_ = 0.08, *p* = .59, Figure 2b).

The result of the PERMANOVA is reflected in the visitation pattern of both pollinators. The most visited plant species by bumblebees throughout the study were *Prunella* sp. (*N* = 624), *Lotus corniculatus* (*N* = 540) and *Trifolium repens* (*N* = 430), which also had relatively high average interaction strengths, meaning they were likely to be visited when present on the plots (Table 1). However, we can find many exceptions of plants species that were only visited by bumblebees on certain plot types. *Prunella* sp. and *Trifolium repens* were mainly interacted with in pastures, whereas *Knautia arvensis* and *Vicia sepium* were two plant species visited on mown plots (Figure 2a). Other plants with high amounts of interactions, such as *Campanula patula* (*N* = 342) or *Ranunculus acris* (*N* = 213), had comparatively low interaction strengths (Table 1). Thus, these plant species were mainly visited on single plots, but hardly on others in which they also occurred. Specifically, *Campanula patula* was visited 308 times on plot AEG22 and *Ranunculus acris* 212 times on plot AEG28, where other frequently visited plants were absent or only available in limited amounts (Figure 2a).

**TABLE 1:** Ten most visited plant species by *Bombus lapidarius* and *Episyrphus balteatus* and the average interaction strength with respective species.

Plant species	*N*	Interaction strength
*Bombus lapidarius*		
*Prunella* sp.	624	0.249
*Lotus corniculatus*	540	0.172
*Trifolium repens*	430	0.247
*Campanula patula*	342	0.052
*Vicia sepium*	244	0.171
*Knautia arvensis*	226	0.254
*Ranunculus acris*	213	0.037
*Trifolium pratense*	198	0.093
*Thymus pulegioides*	165	0.134
*Onobrychis viciifolia*	91	0.108
*Episyrphus balteatus*		
*Galium mollugo*	466	0.329
*Heracleum sphondylium*	151	0.360
*Cerastium holosteoides*	98	0.086
*Thymus pulegioides*	91	0.144
*Ranunculus acris*	59	0.091
*Achillea millefolium*	40	0.074
*Plantago media*	40	0.081
*Stellaria graminea*	32	0.099
*Daucus carota*	24	0.149
*Tragopogon pratensis*	20	0.112

Syrphid flies had the most interactions with *Galium mollugo* (*N* = 466) and *Heracleum sphondylium* (*N* = 151), which were almost always visited when present on the plot (Table 1). Generally, syrphid flies frequently visited certain plant species on different plots independent of management type and LUI, for example as seen for *Galium mollugo*, *Ranunculus acris* or *Plantago media* (Figure 2b). Nevertheless, we also found exceptions such as *Thymus pulegioides*, which was mainly visited on plot AEG49 (*N* = 90) or *Cerastium holosteoides*, which was mainly interacted with on plot AEG05 (*N* = 81). These exceptions, however, did not lead to significant differences in the composition of plant species visited by syrphid flies. Within the 10 most visited plant species, both pollinator species primarily interacted with different plant species and only shared two of them, namely *Thymus pulegioides* and *Ranunculus acris* (Table 1).

### 3.3 Foraging behaviour

Flower constancy was usually high among all individuals of both pollinator species: 67% (187 of 280) of observed bumblebee workers and 65% (167 of 257) of syrphid flies were totally constant, meaning they only visited one plant species during an observation (bumblebees: $\bar{x}$ ± SD: 0.91 ± 0.15; syrphid flies: $\bar{x}$ ± SD: 0.85 ± 0.23). Overall, bumblebees were quicker at handling flowers with an average of 10.3 ± 6.8 ($\bar{x}$ ± SD) flowers visited per minute compared with syrphid flies at 4.5 ± 3.2 ($\bar{x}$ ± SD). Bumblebees also had shorter flight durations ($\bar{x}$ ± SD: 2.7 ± 2.8 s) compared with syrphid flies ($\bar{x}$ ± SD: 5.5 ± 5.4 s), but longer flight distances (bumblebees: $\bar{x}$ ± SD: 91.2 ± 157.3 cm; syrphid flies: $\bar{x}$ ± SD: 63.7 ± 83.6 cm).

**FIGURE A3:**
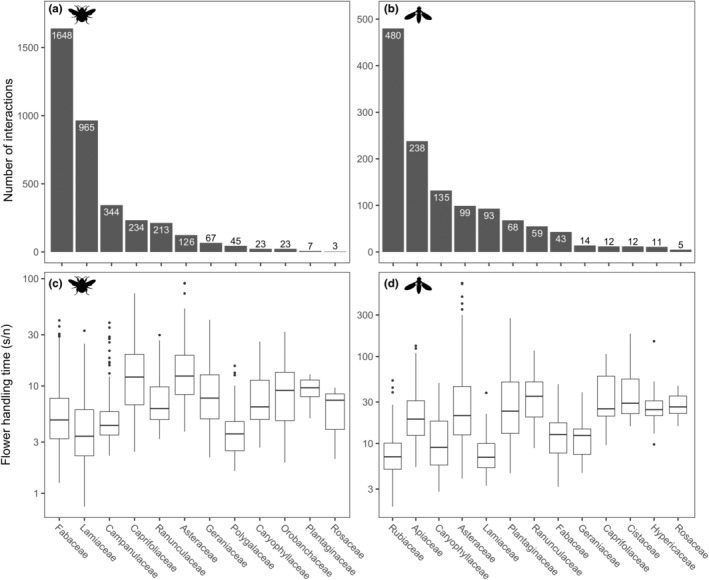
Number of interactions of the bumblebee *Bombus lapidarius* (a) and the syrphid fly *Episyrphus balteatus* (b) per plant family and their respective flower handling times (c, d).

In linear mixed models, ‘plant species’ was consistently a significant predictor of pollinator behaviour regarding the interaction with plants, such as time spent on plant individual, number of flowers visited per plant individual and flower handling time (Table A6), whereas LUI or management type were never significant predictors. As an example, flower handling times varied greatly across the different plant species (Figure A2) or plant families (Figure A3) visited by the pollinators. Otherwise, we found two significant effects for syrphid flies, that is a positive effect of flower diversity on time spent on flowers (LMM_Diversity_: *χ* ^2^ = 5.37, *p* = .02) and flower handling time (LMM_Diversity_: *χ* ^2^ = 15.36, *p* < .001).

**FIGURE 3:**
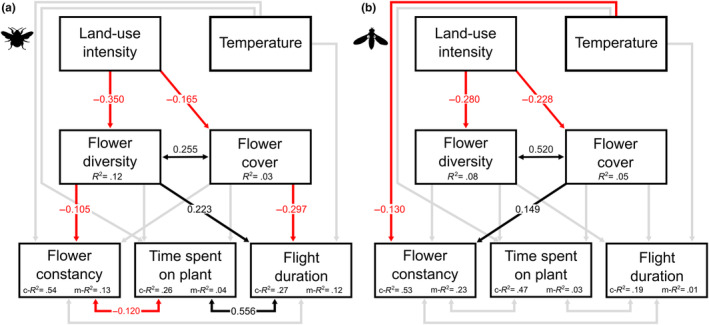
Structural equation models showing the direct and indirect effects of land‐use intensity (LUI) and temperature on pollinator behaviour of the bumblebee *Bombus lapidarius* (a) and the syrphid fly *Episyrphus balteatus* (b). Black lines indicate significant positive, red lines significant negative (*p* < .05) and grey lines non‐significant pathways (*p* > .05). c‐*R* ^2^ = conditional *R* ^2^ (variance explained by both random and fixed effects), m‐*R* ^2^ = marginal *R* ^2^ (variance explained by fixed effects only). See Tables A7 and A8 for additional information.

We conducted structural equation models (SEMs) to reveal possible indirect effects of LUI on pollinator behaviour (Figure 3, see Tables A7 and A8 for detailed results). The analysis showed that LUI reduced flower diversity and flower cover of plant species visited by both bumblebees (Figure 3a, Diversity_LUI_: *β* = −0.350, *p* < .001; Cover_LUI_: *β* = −0.165, *p* = .004) and syrphid flies (Figure 3b, Diversity_LUI_: *β* = −0.280, *p* < .001; Cover_LUI_: *β* = −0.228, *p* < .001). In bumblebees, we found that flower cover reduced flight duration (*β* = −0.297, *p* = .006). In contrast, plant diversity increased bumblebee flight duration (*β* = 0.223, *p* = .02) and decreased flower constancy (*β* = −0.105, *p* = .003). In syrphid flies, temperature reduced flower constancy (*β* = −0.130, *p* = .003), whereas flower cover increased flower constancy (*β* = 0.149, *p* = .003). The global goodness of fit for the models were *χ* ^2^ = 8.147, *p* = .148 and Fisher's *C* = 20.27, *p* = .027 for the bumblebee SEM (Figure 3a); and *χ* ^2^ = 46.462, *p* < .001 and Fisher's *C* = 62.87, *p* < .001 for the syrphid fly SEM (Figure 3b). Correlation error coefficients showed positive correlations between plant diversity and flower cover for both species (bumblebees: *β* = 0.255, *p* < .001; syrphid flies: *β* = 0.520, *p* < .001). For bumblebees, we found a positive correlation between time spent on plant individual and flight duration (*β* = 0.556, *p* < .001) and a negative correlation between time spent on plant individual and flower constancy (*β* = −0.120, *p* = .019).

## 4 DISCUSSION

We conducted behavioural observations of two abundant generalist pollinator species, namely the bumblebee *B. lapidarius* and the syrphid fly *E. balteatus*, in agriculturally managed grasslands and found three major effects of land‐use on patterns of plant–pollinator interactions and pollinator behaviour. (1) The composition, diversity and abundance of plants, used as floral resources by these pollinators changed with LUI and land‐use management type. (2) For bumblebees, but not syrphid flies, we also found these differences in the observed plant–pollinator interactions. (3) Pollinator behaviour was indirectly affected by LUI mediated through limiting floral resource diversity and abundance. Flower diversity and flower cover affected flower constancy and flight duration of the pollinators, with differing effects for the two species.

### 4.1 Floral resources

We found that diversity and abundance of floral resources decreased with LUI (Figure 3), and that plant composition changed along a LUI gradient and with land‐use management types, such as mowing or grazing (Figure 1). A dependency of local plant species composition on land use is in agreement with other studies in the Biodiversity Exploratories showing that the main drivers for plant species diversity loss within managed grasslands are LUI and management practices (in particular mowing and fertilisation) (Busch et al., 2019; Socher et al., 2012). We demonstrated that this is also true when only considering flowering plants that are visited by the investigated pollinators. Thus, intensive land‐use management directly limits floral resources of the pollinators, both in the available species and their overall abundance. Loss of floral resources has long been identified as a primary driver of pollinator decline, with generalists usually being more robust compared to specialists (Peters et al., 2022; Weiner et al., 2014). Both food quantity (flower cover) and quality (polyfloral diet) can affect individual pollinator fitness and foraging, and ultimately decrease pollinator population sizes (Neumüller et al., 2020; Vaudo et al., 2015; Woodard & Jha, 2017). Moreover, recent studies found high‐quality nutrition to buffer for negative effects of other stressors such as pesticides, which are likely to be encountered in nature (Barascou et al., 2021; Klaus et al., 2021; Knauer et al., 2022; Straub et al., 2023). Therefore, promoting floral resources either through land‐use extensification or offering alternative options might be a key factor for pollinators to prevail in agricultural landscapes.

### 4.2 Pollinator–plant interactions

Bumblebees visited certain plant species more frequently when present on the plot (e.g., *Prunella* sp. or *Trifolium repens*, Table 1), whereas others like *Ranunculus acris* (AEG28) or *Campanula patula* (AEG22), were only selected when such options were absent or scarce (Figure 2a). In particular, *Ranunculus* is not a plant genus frequently visited by bumblebee species (Steinbach & Gottsberger, 1995). Adaptive or flexible foraging has often been observed to occur in generalist species such as bumblebees in order to deal with the spatial or temporal variation of floral resources, to avoid competition and to optimise foraging (Bolnick et al., 2003; Heinrich, 1976; Willmer, 2011). Since bumblebees have a local nesting site, they might have limited options in the landscape and thus potentially have to exploit all available resources to optimise foraging. They might also be forced into sub‐optimal or, at least, unusual resource options that can ultimately lead to unfavourable diets (Peters et al., 2022). However, since bumblebees are social insects, resource diversity at the colony level might also buffer negative effects of individually collected, low‐quality food (Brodschneider & Crailsheim, 2010; Moerman et al., 2017). Our results demonstrate that bumblebees can adapt their selection for different host plants depending on floral resource availability, resulting in locally variable visitation patterns shaped by LUI or management practise.

In contrast, syrphid flies showed no difference in the selection of plant species depending on land use. Overall, the solitary syrphid flies might generally be less dependent on plot‐level land‐use effects and associated resource limitation, since they visit plant species, for example from the Apiaceae, Ranunculaceae or Asteraceae (Cowgill et al., 1993; Dunn et al., 2020), which are more robust to land use compared with other species (Busch et al., 2019). This means that syrphids interacted with plant species that were equally distributed over plots independent of LUI and management types. This is particularly reflected in the visitation pattern of *Galium mollugo*, being the most visited plant species and being present on almost every plot (Figure 2b). However, we also found exceptions, such as the interaction with *Thymus pulegioides*, which was almost exclusively visited on plot AEG49, indicating that syrphids might also adapt their visitation patterns if necessary. Moreover, syrphid flies are more mobile and not tied to a local nesting site like the social bumblebees, and thus might be able to switch sites more easily if the habitat or floral availability is not suitable (Almohamad et al., 2009; Doyle et al., 2020).

### 4.3 Pollinator behaviour

The observed individuals of both pollinator species exhibited high levels of flower constancy. A majority of the pollinators only visited one plant species during an observation (mean constancy, bumblebees: 0.91; syrphid flies: 0.85). This behaviour has been described in many studies, especially for bumblebees (Free, 1970; Gegear & Laverty, 2005; Heinrich, 1976), but also for syrphid flies (Goulson & Wright, 1998). However, pollen analysis techniques have recently shown constancy in bumblebees not to be as high as previously described. For example, bumblebees carry pollen loads with an average diversity of 2.5 pollen types (Yourstone et al., 2023) or visit about five plant species (Martínez‐Bauer et al., 2021) per foraging trip. Observations of pollinator individuals in our study were conducted on a small spatial scale and lasted only for several minutes, not reflecting the spectrum of a whole foraging trip. Our results suggest that bumblebees and syrphid flies are highly constant when foraging, at least on a plot‐level scale, but can potentially visit different plants when switching sites.

We found indirect, but no direct, effects of LUI on foraging behaviour. Behavioural variables regarding plant–pollinator interactions, such as flower handling times, were mostly dependent on plant species identity rather than land‐use (Figure A2, Table A6). Different plant species have different flower morphologies requiring different handling techniques by the pollinators to access to the floral rewards (Lewis, 1993; Willmer, 2011). This probably explains the strong dependency of pollinator behaviour on the visited plant species and was a factor that we could not exclude when making observations in the field. Future studies might shed more light on the effects of land‐use on plant‐specific pollinator behaviour by experimentally testing how land‐use shapes floral traits, such as pollen and nectar rewards or scent of the visited plant species, and how these traits, in turn, affect pollinator foraging.

SEMs revealed indirect effects of LUI on pollinator behaviour by reducing local flower diversity and cover. In bumblebees, flower cover had a negative effect on flight duration, meaning that flights were longer when flower cover was lower. Thus, the bumblebees were likely to forage energetically less efficient when visiting high LUI plots by taking more time to fly from one plant individual to another. This might ultimately result in smaller pollen loads, which in turn affects larval nutrition and body size (Carvell et al., 2012; Kendall et al., 2019). Straub et al., 2022 have studied *B. lapidarius* in the Schwäbische Alb Exploratory and found that bumblebee workers are smaller on plots with higher LUI, a result that could potentially be explained by our findings.

In contrast, plant diversity increased bumblebee flight duration and decreased flower constancy. Pollinators that forage only on certain of the available plant species may have longer foraging flights on high plant diversity plots since they might take more time to find their preferred resource (Bar‐Shai et al., 2004). Moreover, such sites offer more options for the pollinators in terms of available floral resources and may thus lead to reduced flower constancy, that is increase the likelihood to switch plant species between floral visits, especially when flight durations are already long (Raine & Chittka, 2007). The exploitation of alternative options might be beneficial for the pollinators enabling them to receive a balanced nutrition in landscapes with limited resources (Ruedenauer et al., 2016). Higher flower diversity on land‐use extensive plots may thus decrease foraging efficiency of bumblebees due to longer foraging flights, which might be compensated by an increase of overall resource options and nutritional intake.

In syrphid flies, we detected a positive effect of flower cover on flower constancy. Flower constancies of bumblebees were shown to increase when the resource abundance is higher (Gegear & Thomson, 2004; Hayes & Grüter, 2023), meaning they are more likely to visit the same preferred plant species if those species are available in higher densities. Here, this might also apply to syrphid flies, particularly since the frequently visited plant species *Galium mollugo* was abundant on most of the plots. Additionally, we detected an effect of temperature reducing the flower constancy of syrphid flies, while we found no temperature effect for bumblebees. Reasons for a change of behaviour might either be due to a change in plant attractiveness (Höfer et al., 2020) or in pollinator physiology like foraging outside the thermal optimum (McCallum et al., 2013). Overall, we found that local LUI played a smaller role in determining the foraging behaviour of syrphid flies as compared to bumblebees. Other studies have also shown that syrphid flies do not react as strongly, or even opposite, to land‐use change at a landscape level compared with bees (Jauker et al., 2009).

## 5 CONCLUSION

We have demonstrated that intensive land use and its practices in agricultural grasslands can reduce resource availability for the two generalist pollinator species studied here, *B. lapidarius* and *E. balteatus*. For bumblebees, but not syrphid flies, we have also found differences in the observed flower visitation patterns depending on land‐use. This means that bumblebees adapt their selection of host plants depending on their availability, which might optimise foraging but potentially force them into sub‐optimal resource options. Such changes in floral compositions might be even more severe for specialist species (oligolects), which are dependent on single plant species or genera (Weiner et al., 2011). The foraging behaviour of bumblebees also responded more strongly to the indirect effects of land‐use, namely the reduction in local plant diversity and abundance. This possibly decreases their foraging efficiency or nutritional intake when foraging on high LUI sites, which might further contribute to the decline of pollinator populations in an already unstable environment. Therefore, the maintenance or compensation of local plant diversity and abundance in agricultural grasslands is important in order to conserve locally bound pollinator species. In syrphid flies, plant visitation patterns and foraging behaviour were generally less affected by local land‐use compared with bumblebees, probably because of their preference for plant species that are not that vulnerable to land‐use change. Furthermore, syrphid flies are more mobile, that is they are not dependent on a local nesting site and thus might perform better within agricultural landscapes.

## AUTHOR CONTRIBUTIONS

**Markus Birkenbach:** Conceptualization (equal); data curation (lead); formal analysis (lead); investigation (equal); methodology (equal); visualization (lead); writing – original draft (lead); writing – review and editing (equal). **Florian Straub:** Conceptualization (equal); data curation (equal); methodology (equal); writing – review and editing (supporting). **Anna Kiesel:** Data curation (equal); investigation (equal); methodology (equal). **Lena Wilfert:** Conceptualization (equal); formal analysis (equal); funding acquisition (equal); methodology (equal); project administration (equal); writing – original draft (supporting); writing – review and editing (equal). **Manfred Ayasse:** Conceptualization (equal); funding acquisition (equal); methodology (equal); project administration (equal); resources (equal); writing – original draft (supporting); writing – review and editing (equal). **Jonas Kuppler:** Conceptualization (lead); data curation (equal); formal analysis (equal); funding acquisition (equal); methodology (lead); project administration (equal); resources (equal); writing – original draft (supporting); writing – review and editing (equal).

## CONFLICT OF INTEREST STATEMENT

All authors declare no competing interests.

## Data Availability

All data are available in the Biodiversity Exploratories data repository BExIS (https://www.bexis.uni‐jena.de/) with the accession numbers 31495 (plant data), 31494 (behavioural data) and 31686 (R script). All data are permanently archived in BExIS and will be publicly available after an embargo period of 5 years from the end of the data assembly.

## APPENDIX 1

### METHODOLOGY OF MEAN CALCULATION OF CATEGORICAL VARIABLES

Flower cover was recorded in four categories: <1%, 1%–10%, 10%–40% and >40%. For the middle categories, we calculated means by adding the upper and the lower limit (e.g. 1 + 10 = 11) and then dividing this number by two. The lowest category (<1%) was set to 0.5 (as 0 + 1 = 0.5). For the highest category (>40%), we halved the difference of the previous interval ((40 − 10)/2 = 15) and added this to the value (40 + 15 = 55). Accordingly, the final flower cover values that were used were 0.5, 5.5, 25 and 55. To assess flower cover of an individual plant species on a plot, we multiplied total flower cover with the individual flower cover, e.g. if the individual flower cover of *Trifolium pratense* on a plot was >40% and total flower cover was 10%–40%, we calculated 55 × (25/100) = 13.75.

Mean calculation of the categorical variables number of flowers visited per plant individual and flight distance was carried out according to the flower cover and resulted in the values 1, 2.5, 4.5, 6.5, 9 and 11 for the number of flowers visited and 5, 20, 45, 80, 150, 300, 550, 850 and 1150 cm for the flight distance.

**TABLE A1:** Land‐use information, number of pollinator individuals observed and visitations per plot.

PlotID	G_STD	M_STD	F_STD	LUI	Number of observations		Number of visits	Management type
					*Bombus lapidarius*	*Episyrphus balteatus*		
AEG01	0	1.47	4.39	2.42		8	1	Meadow
AEG02	0	2.94	4.65	2.75		11	1	Meadow
AEG03	0	1.47	0	1.21	17	10	2	Meadow
AEG04	0.68	0.74	0.72	1.46		8	1	Mown pasture
AEG05	3.05	0.74	0.86	2.16		14	1	Mown pasture
AEG06	1	0.74	0.86	1.61		12	1	Mown pasture
AEG07	1.06	0	0	1.03	8	9	2	Pasture
AEG08	0.13	1.47	0	1.26	14		2	Mown pasture
AEG09	1.09	0	0	1.05	9	3	1	Pasture
AEG10	0.12	1.47	0.18	1.33	4	4	1	Mown pasture
AEG11	0.74	1.47	2.84	2.25		11	1	Mown Pasture
AEG14	0	1.47	3.72	2.28	17		1	Meadow
AEG15	0	2.21	3.9	2.47		13	1	Meadow
AEG16	5.51	0	0.61	2.47	13	4	3	Pasture
AEG17	0	1.47	0	1.21	10		1	Meadow
AEG18	0	2.21	1.56	1.94		19	1	Meadow
AEG19	1.49	0	0.72	1.49		10	1	Pasture
AEG20	2.13	0	0	1.46		10	1	Pasture
AEG21	6.04	0.74	0.61	2.72	6	13	2	Mown pasture
AEG22	0	1.47	0.88	1.53	15	12	2	Meadow
AEG23	0	2.21	0	1.49	7	13	2	Meadow
AEG24	0.57	2.21	1.19	1.99	2	13	1	Mown pasture
AEG25	0.53	0	0	0.72	17	10	2	Pasture
AEG26	4.35	0	0	2.09	12		1	Pasture
AEG27	2.26	0	0	1.5	10		1	Pasture
AEG28	0.66	0	0	0.81	17		1	Pasture
AEG29	1	0.74	0.4	1.46	13	1	1	Mown pasture
AEG30	0	2.21	1.5	1.93	15	6	2	Meadow
AEG31	0	1.47	0	1.21	16		1	Meadow
AEG32	0.34	0	0	0.58	14	9	3	Pasture
AEG33	0.57	0	0	0.75	12	2	2	Pasture
AEG34	1.12	0	0	1.06	12	13	2	Pasture
AEG38	0.43	1.47	0.36	1.51		8	1	Mown pasture
AEG39	0	1.47	2.45	1.98		12	1	Meadow
AEG40	0.97	0.74	0.69	1.55		15	1	Mown pasture
AEG41	0	2.21	2.75	2.23		13	1	Meadow
AEG42	0	2.21	2.39	2.14		2	1	Meadow
AEG43	1.48	0	0	1.22	7	7	2	Pasture
AEG44	3.16	0	0	1.78		6	2	Pasture
AEG45	0.39	1.47	0.4	1.5	7	2	1	Mown pasture
AEG46	5.29	0	0	2.3	9		1	Pasture
AEG48	1.01	0	0	1.01	12		1	Pasture
AEG49	1.69	0	0	1.3	12	12	2	Pasture
					∑ 307	∑ 315		

*Note*: Number of visits: number of times the plots were visited by the researchers.

Abbreviations: F_STD, fertilization intensity; G_STD, grazing intensity; LUI, land‐use intensity index; M_STD, mowing intensity.

**TABLE A2:** Behavioural parameters recorded in categories used as modifier in the coding scheme of the software Observer XT.

Behavioural parameters	Categories		
Plant species	*Achillea millefolium*	*Genista sagittalis*	*Potentilla reptans*
	*Anthriscus sylvestris*	*Geranium pratense*	*Prunella* sp.
	*Anthyllis vulneraria*	*Geranium pyrenaicum*	*Ranunculus acris*
	*Bellis perennis*	*Geranium sylvaticum*	*Rhinanthus alectorolophus*
	*Caduus crispus*	*Geum rivale*	*Rhinanthus minor*
	*Campanula patula*	*Helianthemum nummularium*	*Salvia pratensis*
	*Campanula rotundifolia*	*Heracleum sphondylium*	*Sanguisorba minor*
	*Carum carvi*	*Hippocrepis comosa*	*Scabiosa columbaria*
	*Centaurea jacea*	*Hypericum perforatum*	*Silene vulgaris*
	*Cerastium holosteoides*	*Hypochaeris radicata*	*Stellaria graminea*
	*Chaerophyllum aureum*	*Knautia arvensis*	*Taraxacum* sp.
	*Cirsium acaule*	*Leucanthemum vulgare*	*Teuricum chamaedrys*
	*Cirsium arvense*	*Lotus corniculatus*	*Teuricum montanum*
	*Cirsium eriophorum*	*Medicago lupulina*	*Thymus pulegioides*
	*Cirsium palustre*	*Onobrychis viciifolia*	*Tragopogon pratensis*
	*Cirsium vulgare*	*Ononis spinosa*	*Trifolium medium*
	*Clinopodium vulgare*	*Picris hieracioides*	*Trifolium pratense*
	*Conopodium majus*	*Pilosella officinarum*	*Trifolium repens*
	*Crepis biennis*	*Plantago lanceolata*	*Veronica austriaca*
	*Daucus carota*	*Plantago major*	*Veronica chamaedrys*
	*Dianthus carthusianorum*	*Plantago media*	*Vicia sativa*
	*Galium mollugo*	*Polygala comosa*	*Vicia sepium*
	*Galium verum*	*Potentilla erecta*	
Number of flowers visited per plant individual	1	6–7	
	2–3	8–10	
	4–5	>10	
Flight distance	<10 cm	61–100 cm	401–700 cm
	10–30 cm	101–200 cm	701–1000 cm
	31–60 cm	201–400 cm	>1000 cm

**TABLE A3:** Number of floral interactions (*N*) of the bumblebee *Bombus lapidarius* per plant species with mean and standard deviation of behavioural parameters.

Plant species	*N*	Flowers visited per plant individual $\bar{x}$(*n*) ± SD	Time spent on plant individual $\bar{x}$(*s*) ± SD	Flower handling time $\bar{x}$(*s*/*n*) ± SD	Interaction strength
*Anthyllis vulneraria*	20	4.45 ± 2.23	30.26 ± 11.12	7.69 ± 3.5	0.303
*Bellis perennis*	1	1	3.75	3.75	0.001
*Campanula patula*	342	1.07 ± 0.32	5.91 ± 5.34	5.45 ± 4.24	0.052
*Campanula rotundifolia*	2	1	3.25 ± 0.47	3.25 ± 0.47	0.002
*Carduus crispus*	3	4.83 ± 4.01	82.36 ± 70.78	17.12 ± 1.22	0.111
*Centaurea jacea*	4	1	22.69 ± 5.94	22.69 ± 5.94	0.045
*Cerastium holosteoides*	8	1	6.57 ± 2.39	6.57 ± 2.39	0.024
*Cirsium arvense*	1	1	15.12	15.12	0.005
*Cirsium eriophorum*	3	1.5 ± 0.87	54.61 ± 34.48	44.14 ± 39.86	0.074
*Cirsium palustre*	15	2.2 ± 1.57	46.37 ± 28.36	29.22 ± 25.05	0.189
*Cirsium vulgare*	5	1.3 ± 0.67	44.37 ± 30.38	32.86 ± 10.08	0.026
*Clinopodium vulgare*	1	2.5	10.1	4.04	0.005
*Crepis biennis*	2	1	25.53 ± 15.57	25.53 ± 15.57	0.003
*Genista sagaittalis*	27	2.02 ± 0.88	17.56 ± 10.52	9.27 ± 5.6	0.04
*Geranium pratense*	54	1.92 ± 1.28	15.7 ± 11.53	9.97 ± 8.77	0.205
*Geranium sylvaticum*	13	1.58 ± 0.76	20.81 ± 8.25	14.69 ± 7.37	0.025
*Geum rivale*	2	1	8.52 ± 1.62	8.52 ± 1.62	0.02
*Hippocrepis comosa*	83	1.92 ± 1.19	10.01 ± 7.13	5.53 ± 2.51	0.158
*Hypochaeris radicata*	71	1	11.38 ± 6.43	11.38 ± 6.43	0.036
*Knautia arvense*	225	1.1 ± 0.38	15.82 ± 13.46	14.24 ± 9.88	0.254
*Lotus corniculatus*	540	2.67 ± 1.4	12.45 ± 10.05	5.09 ± 3.83	0.172
*Medicago lupulina*	6	2.58 ± 1.11	13.03 ± 3.19	5.73 ± 2.46	0.01
*Onobrychis viciifolia*	91	2.85 ± 1.75	12.13 ± 10.55	4.94 ± 4.8	0.108
*Ononis spinosa*	4	1.75 ± 0.87	6.6 ± 2.31	4.4 ± 1.95	0.025
*Origanum vulgare*	46	3.51 ± 1.36	21.47 ± 11.7	6.12 ± 2.31	0.074
*Picris hieracioides*	1	2.5	9.97	3.99	0.019
*Pilosella officinarum*	3	1	21.24 ± 10.22	21.24 ± 10.22	0.002
*Plantago media*	2	1	11.38 ± 0.84	11.38 ± 0.84	0.001
*Polygala comosa*	45	2.18 ± 0.74	8.98 ± 6.2	4.45 ± 3.15	0.057
*Potentilla reptans*	1	4.5	9.43	2.1	0.004
*Prunella* sp.	624	2.6 ± 1.29	9.73 ± 8.64	4.03 ± 3.09	0.249
*Ranunculus acris*	213	1.01 ± 0.15	8.31 ± 5.2	8.25 ± 5.21	0.037
*Rhinanthus alectorolophus*	9	2 ± 0.75	30.77 ± 6.61	17.67 ± 8.17	0.023
*Rhinanthus minor*	14	2.04 ± 1.26	11.16 ± 9.14	5.65 ± 2.82	0.036
*Salvia pratensis*	21	3.31 ± 2.32	26.57 ± 13.36	10.01 ± 6.56	0.023
*Scabiosa columbaria*	8	1	39.59 ± 23.11	39.59 ± 23.11	0.148
*Silene vulgaris*	14	2.18 ± 1.42	18.57 ± 14.94	10.42 ± 7.4	0.08
*Stellaria graminea*	1	1	6.32	6.32	0.001
*Teucrium chamaedrys*	43	3.33 ± 1.89	14.66 ± 10.05	4.88 ± 2.98	0.198
*Teucrium montanum*	65	3.1 ± 2.51	14.28 ± 10.87	5.52 ± 3.22	0.147
*Thymus pulegioides*	165	3.45 ± 2.32	16.51 ± 15.82	5.37 ± 3.53	0.134
*Tragopogon pratensis*	15	1	17.43 ± 8.34	17.43 ± 8.34	0.02
*Trifolium pratense*	196	3.07 ± 2.79	18.77 ± 15.19	8.07 ± 5.48	0.093
*Trifolium repens*	430	1.46 ± 1.06	9.64 ± 7.5	6.81 ± 3.44	0.247
*Veronica austriaca*	5	3.3 ± 1.1	26.88 ± 6.78	8.68 ± 2.87	0.003
*Vicia sativa*	1	2.5	22.53	9.01	0.023
*Vicia sepium*	242	2.88 ± 1.41	13.68 ± 10.35	5.47 ± 4.69	0.171

*Note*: Interaction strength represents the average preference for a plant species when present on a plot.

**TABLE A4:** Number of floral interactions (*N*) of the syrphid fly *Episyrphus balteatus* per plant species with mean and standard deviation of behavioural parameters.

Plant species	*N*	Flowers visited per plant individual $\bar{x}$(*n*) ± SD	Time spent on plant individual $\bar{x}$(*s*) ± SD	Flower handling time $\bar{x}$(*s*/*n*) ± SD	Interaction strength
*Achillea millefolium*	40	3.9 ± 3.16	54.47 ± 58.35	14.34 ± 6.13	0.074
*Anthriscus sylvestris*	28	7.7 ± 3.96	148.39 ± 121.77	18.52 ± 9.68	0.341
*Anthyllis vulneraria*	23	2.17 ± 2.33	33.28 ± 45.46	16.61 ± 11.9	0.535
*Carum carvi*	18	4.64 ± 1.81	62.41 ± 24.91	14.48 ± 6.61	0.075
*Cerastium hololsteoides*	98	1.12 ± 0.41	12.71 ± 9.74	11.7 ± 8.71	0.086
*Chaerophyllum aureum*	14	5.46 ± 3.95	69.07 ± 51.25	14.94 ± 12.51	0.435
*Conopodium majus*	4	4.75 ± 3.28	131.86 ± 133.43	22.61 ± 10.43	0.100
*Crepis biennis*	9	1.33 ± 0.66	53.32 ± 60.01	50.36 ± 61.99	0.022
*Daucus carota*	24	4.71 ± 3.44	147.72 ± 234.17	22.43 ± 19.17	0.149
*Dianthus carthusianorum*	5	1	28.74 ± 16.71	28.74 ± 16.71	0.111
*Galium mollugo*	466	5.06 ± 3.23	43.12 ± 49.06	8.33 ± 5.26	0.329
*Galium verum*	14	6.07 ± 3.31	42.73 ± 37.25	6.58 ± 2.65	0.058
*Geranium pyrenaicum*	14	1.36 ± 0.99	17.39 ± 12.39	14.28 ± 9.41	0.060
*Helianthemum nummularium*	12	1	50.3 ± 49.64	50.3 ± 49.64	0.239
*Heracleum sphondylium*	151	6.26 ± 3.45	186.27 ± 181.93	28.94 ± 21.47	0.360
*Hypericum perforatum*	11	3.82 ± 3.32	144.33 ± 195.38	36.6 ± 39.14	0.056
*Hypochaeris radicata*	8	1.38 ± 0.69	93.18 ± 167.65	48.58 ± 63	0.036
*Knautia arvense*	9	1	46.92 ± 34.41	46.92 ± 34.41	0.030
*Leucanthemum vulgare*	1	1	17.59	17.59	0.002
*Lotus corniculatus*	2	1	12.67 ± 0.94	12.67 ± 0.94	0.003
*Origanum vulgare*	2	6.75 ± 6.01	43.29 ± 45.57	5.64 ± 1.73	0.007
*Pilosella officinarum*	13	1	54.53 ± 56.79	54.53 ± 56.79	0.069
*Plantago lanceolata*	14	1	105.98 ± 88.35	105.98 ± 88.35	0.034
*Plantago major*	13	1	60.15 ± 73.88	60.15 ± 73.88	0.045
*Plantago media*	40	1.58 ± 1.76	37.36 ± 44.37	26.73 ± 32.19	0.081
*Potentilla erecta*	4	1	24.89 ± 8.11	24.89 ± 8.11	0.103
*Ranunculus acris*	55	1.19 ± 0.5	43.58 ± 26.66	40.02 ± 26.69	0.091
*Sanguisorba minor*	1	1	46.13	46.13	0.023
*Scabiosa columbaria*	3	1	22.26 ± 4.9	22.26 ± 4.9	0.200
*Stellaria graminea*	29	1.31 ± 0.62	16.3 ± 9.87	13.27 ± 7.92	0.099
*Taraxacum* sp.	8	1	295.41 ± 229.18	295.41 ± 229.18	0.243
*Thymus pulegioides*	91	2.93 ± 1.9	21.1 ± 12.24	8.45 ± 5.13	0.144
*Tragopogon pratensis*	20	1	110.25 ± 168.43	110.25 ± 168.43	0.112
*Trifolium medium*	3	1	11.2 ± 4.64	11.2 ± 4.64	0.038
*Trifolium repens*	13	1.69 ± 0.78	18.16 ± 9.46	11.65 ± 5.67	0.016
*Veronica chamaedrys*	1	1	34.46	34.46	0.100
*Vicia sepium*	2	1	17.2 ± 0.88	17.2 ± 0.88	0.005

*Note*: Interaction strength represents the average preference for a plant species when present on a plot.

**TABLE A5:** Separation of plant species along the two NMDS axes.

*Bombus lapidarius*				*Episyrphus balteatus*			
Plant species	NMDS1	NMDS2	*p*‐Value	Plant species	NMDS1	NMDS2	*p*‐Value
*Prunella* sp.	0.697	0.185	**<.001**	*Hyp. Radicata*	0.729	0.100	**<.001**
*Teuc.montanum*	0.554	0.065	**.005**	*Pil. Officinarum*	0.684	0.113	**<.001**
*Pil. officinarum*	0.515	−0.388	**.002**	*Hyp. Perforatum*	0.651	0.005	**<.001**
*Polygala comosa*.	0.413	−0.102	.086	*Thymus pulegioides*	0.624	0.005	**<.001**
*Thymus pulegioides*	0.409	−0.195	.063	Rare species	0.597	0.061	**.001**
*Hyp. radicata*	0.392	−0.212	.074	*Lotus corniculatus*	0.561	0.127	**.002**
*Genista sagittalis*	0.359	−0.541	**.001**	*Galium verum*	0.556	−0.034	**.002**
*Lotus corniculatus*	0.344	−0.063	.211	*H. nummularium*	0.517	0.116	**.005**
*Veronica austriaca*	0.320	−0.444	**.014**	*Origanum vulgare*	0.425	−0.008	**.044**
*Hippocrepis comosa*	0.182	−0.364	.117	*Leuc. Vulgare*	0.359	0.305	**.020**
*Origanum vulgare*	0.165	−0.124	.596	*Daucus carota*	0.358	0.013	.119
*Trifolium repens*	0.076	0.427	.077	*Knautia arvensis*	0.207	0.318	.094
*Ranunculus acris*	0.047	−0.084	.894	*Ach. Millefolium*	0.175	−0.361	.067
*C. holosteoides*	0.037	0.063	.938	*Ranunculus acris*	0.156	0.251	.236
*Plantago media*	−0.009	0.456	.064	*Tra. Pratensis*	0.112	0.143	.594
*Campanula patula*	−0.029	−0.055	.971	*Carum carvi*	0.107	−0.254	.304
*Onobrychis viciifolia*	−0.046	−0.083	.929	*Crepis biennis*	0.048	0.351	.126
*Stellaria graminea*	−0.063	0.262	.416	*Vicia sepium*	0.012	0.342	.143
*Tra. pratensis*	−0.104	0.440	.064	*Trifolium repens*	0.007	−0.225	.444
*Salvia pratensis*	−0.183	−0.528	**.011**	*Stellaria graminea*	−0.039	0.016	.972
Rare species	−0.215	−0.397	.066	*Plantago major*	−0.104	−0.251	.311
*Knautia arvensis*	−0.217	0.249	.261	*Plantago media*	−0.106	−0.280	.237
*Ger. sylvaticum*	−0.219	−0.195	.351	*H. sphondylium*	−0.207	−0.258	.169
*R. alectorolophus*	−0.245	0.087	.453	*Plantago lanceolata*	−0.249	−0.352	**.043**
*Trifolium pratense*	−0.252	0.228	.227	*Galium mollugo*	−0.295	0.222	.103
*Crepis biennis*	−0.341	−0.125	.182	*C. holosteoides*	−0.435	−0.119	**.028**
	−0.564	−0.194	**.005**				

*Note*: Plant species were sorted by the first axis, which is mostly responsible for the separation of pastures (positive values) from mown plots (negative values). Significant contributions are in bold.

**TABLE A6:** Results of linear mixed effect models (LMMs) of behavioural response variables regarding interactions with flowering plants.

	*Bombus lapidarius* (*N* = 3698)			*Episyrphus balteatus* (*N* = 1269)		
Response	log(Time spent on plant individual)					
Predictors	*χ* ^2^	df	*p*‐Value	*χ* ^2^	df	*p*‐Value
LUI	0.83	1	.36	0.08	1	.84
Management type	0.48	2	.79	1.32	2	.57
Flower diversity	1.45	1	.23	4.91	1	**.03**
Flower cover	1.71	1	.19	0.06	1	.83
Temperature	0.28	1	.6	4.95	1	**.03**
Plant species	123.30	47	**<.001**	319.18	36	**<.001**
		c‐*R* ^2^: .63	m‐*R* ^2^: .1		c‐*R* ^2^: .54	m‐*R* ^2^: .35

Response	sqrt(Numbers of flowers visited)					
Predictors	*χ* ^2^	df	*p*‐Value	*χ* ^2^	df	*p*‐Value
LUI	0.68	1	.41	0.07	1	.79
Management type	0.31	2	.86	1.57	2	.58
Flower diversity	0.16	1	.69	1.57	1	.21
Flower cover	0.07	1	.79	0.37	1	.54
Temperature	3.16	1	.08	3.03	1	.08
Plant species	311.76	47	**<.001**	509.41	36	**<.001**
		c‐*R* ^2^: .58	m‐*R* ^2^: .25		c‐*R* ^2^: .57	m‐*R* ^2^: .41

Response	log(Flower handling time)					
Predictors	*χ* ^2^	df	*p*‐Value	*χ* ^2^	df	*p*‐Value
LUI	0.57	1	.45	0.02	1	.88
Management type	1.4	2	.5	0.84	2	.67
Flower diversity	0.88	1	.35	14.9	1	**<.001**
Flower cover	2.59	1	.11	0.96	1	.33
Temperature	0.96	1	.32	1.46	1	.23
Plant species	272.69	47	**<.001**	690.99	36	**<.001**
		c‐*R* ^2^: .72	m‐*R* ^2^: .21		c‐*R* ^2^: .64	m‐*R* ^2^: .51

*Note*: Behavioural variables are mostly dependent on plant species and not land‐use intensity (LUI) or management types. Significant effects are in bold. “Pollinator individual” nested in “plot” was used as a random factor. c‐*R* ^2^ = conditional *R* ^2^ (variance explained by both random and fixed effects); m‐*R* ^2^ = marginal *R* ^2^ (variance explained by fixed effects only).

**TABLE A7:** Results of structural equation modelling (SEM) analysing the indirect effect of LUI and direct effect of temperature on the behaviour of the bumblebee *Bombus lapidarius*.

SEM – *Bombus lapidarius* (*N* = 280)						
Global goodness of fit	*χ* ^2^ = 8.147, *p* = .148, df = 5			Fisher's *C* = 20.265, *p* = .027, df = 10		
Response		Predictor	Estimate	Std. error	*p*‐Value	Std. estimate
Flower diversity	←	LUI	−0.191	0.03	**<.001**	−0.35
Flower cover	←	LUI	−6.97	2.43	**.004**	−0.165
Time spent on plant	←	Flower diversity	0.268	0.189	.175	0.13
	←	Flower cover	−0.005	0.003	.051	−0.198
	←	Temperature	0.002	0.019	.924	0.01
Flight duration	←	Flower diversity	0.431	0.174	**.019**	0.223
	←	Flower cover	−0.007	0.002	**.005**	−0.297
	←	Temperature	0.027	0.017	.122	0.157
Flower constancy	←	Flower diversity	−0.702	0.235	**.003**	−0.105
	←	Flower cover	−0.0045	0.004	.244	−0.053
	←	Temperature	−0.27	0.027	.319	−0.046
Correlation error coefficients						
Flower diversity	↔	Flower cover	0.255	–	**<.001**	0.255
Time spent on plant	↔	Flight duration	0.556	–	**<.001**	0.556
Flower constancy	↔	Time spent on plant	−0.12	–	**.02**	−0.12
Flower constancy	↔	Flight duration	−0.087	–	.068	−0.087
Individual *R* ^2^	m‐*R* ^2^		c‐*R* ^2^			
Flower diversity	.12		–			
Flower cover	.03		–			
Time spent on plant	.04		.26			
Flight duration	.12		.27			
Flower constancy	.13		.54			

*Note*: Significant effects are in bold. “Plot” and “plant species” were used as a random factor whenever appropriate. c‐*R* ^2^ = conditional *R* ^2^ (variance explained by both random and fixed effects); m‐*R* ^2^ = marginal *R* ^2^ (variance explained by fixed effects only).

**TABLE A8:** Results of structural equation modelling (SEM) analysing the indirect effect of LUI and direct effect of temperature on the behaviour of the syrphid fly *Episyrphus balteatus*.

SEM – *Episyrphus balteatus* (*N* = 257)						
Global goodness of fit	*χ* ^2^ = 46.462, *p* < .001, df = 5			Fisher's *C* = 62.865, *p* < .001, df = 10		
Response		Predictor	Estimate	Std. error	*p*‐Value	Std. estimate
Flower diversity	←	LUI	−0.169	0.036	**<.001**	−0.28
Flower cover	←	LUI	−7.504	1.996	**<.001**	−0.228
Time spent on plant	←	Flower diversity	0.329	0.224	.158	0.132
	←	Flower cover	−0.003	0.004	.562	−0.055
	←	Temperature	−0.027	0.02	.206	−0.101
Flight duration	←	Flower diversity	0.116	0.168	.498	0.071
	←	Flower cover	−0.003	0.003	.45	−0.082
	←	Temperature	0.017	0.016	.319	0.096
Flower constancy	←	Flower diversity	0.148	0.18	.412	0.034
	←	Flower cover	0.018	0.004	**.003**	0.149
	←	Temperature	−0.059	0.019	**.002**	−0.13
Correlation error coefficients						
Flower diversity	↔	Flower cover	0.52	–	**<.001**	0.52
Time spent on plant	↔	Flight duration	0.077	–	.109	0.077
Flower constancy	↔	Time spent on plant	–0.068	–	.137	–0.068
Flower constancy	↔	Flight duration	0.017	–	.392	0.017
Individual *R* ^2^	m‐*R* ^2^		c‐*R* ^2^			
Flower diversity	.08		–			
Flower cover	.05		–			
Time spent on plant	.03		.47			
Flight duration	.01		.19			
Flower constancy	.23		.53			

*Note*: Significant effects are in bold. “Plot” and “plant species” were used as a random factor whenever appropriate. c‐*R* ^2^ = conditional *R* ^2^ (variance explained by both random and fixed effects); m‐*R* ^2^ = marginal *R* ^2^ (variance explained by fixed effects only).
